# Supplementary material for: Development, Processing and Applications of a UV-Curable Polymer with Surface Active Thiol Groups
Source: Nanomaterials (Basel). 2020 Sep 14;10(9):1829. doi: 10.3390/nano10091829 (PMC7558128; doi:10.3390/nano10091829)
Supplement: Supplementary file 1 [file nanomaterials-10-01829-s001.pdf]

Development, Processing and Applications of a UV-Curable Polymer with Surface Active Thiol Groups

Manuel Müller *, Rukan Nasri, Lars Tiemann and Irene Fernandez-Cuesta *

Center for Hybrid Nanostructures (CHyN), Institut für Nanostruktur- und Festkörperphysik (INF), Universität Hamburg, Luruper Chaussee 149, Hamburg, 22761, Germany

* Correspondence: mamuelle@physnet.uni-hamburg.de, ifernand@physnet.uni-hamburg.de

Supplementary Information

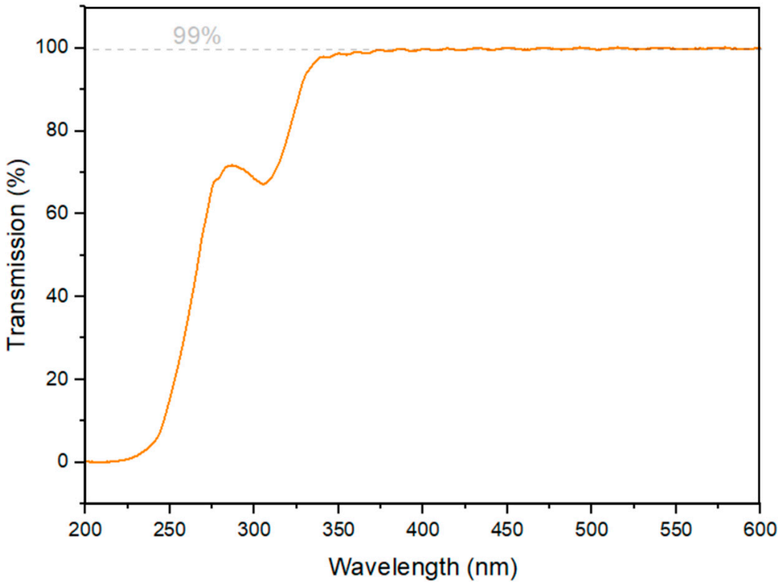

Figure S1. *Polymer transparency.* Transmission of light through a film (1.6 μm thick) of cured thiol polymer.

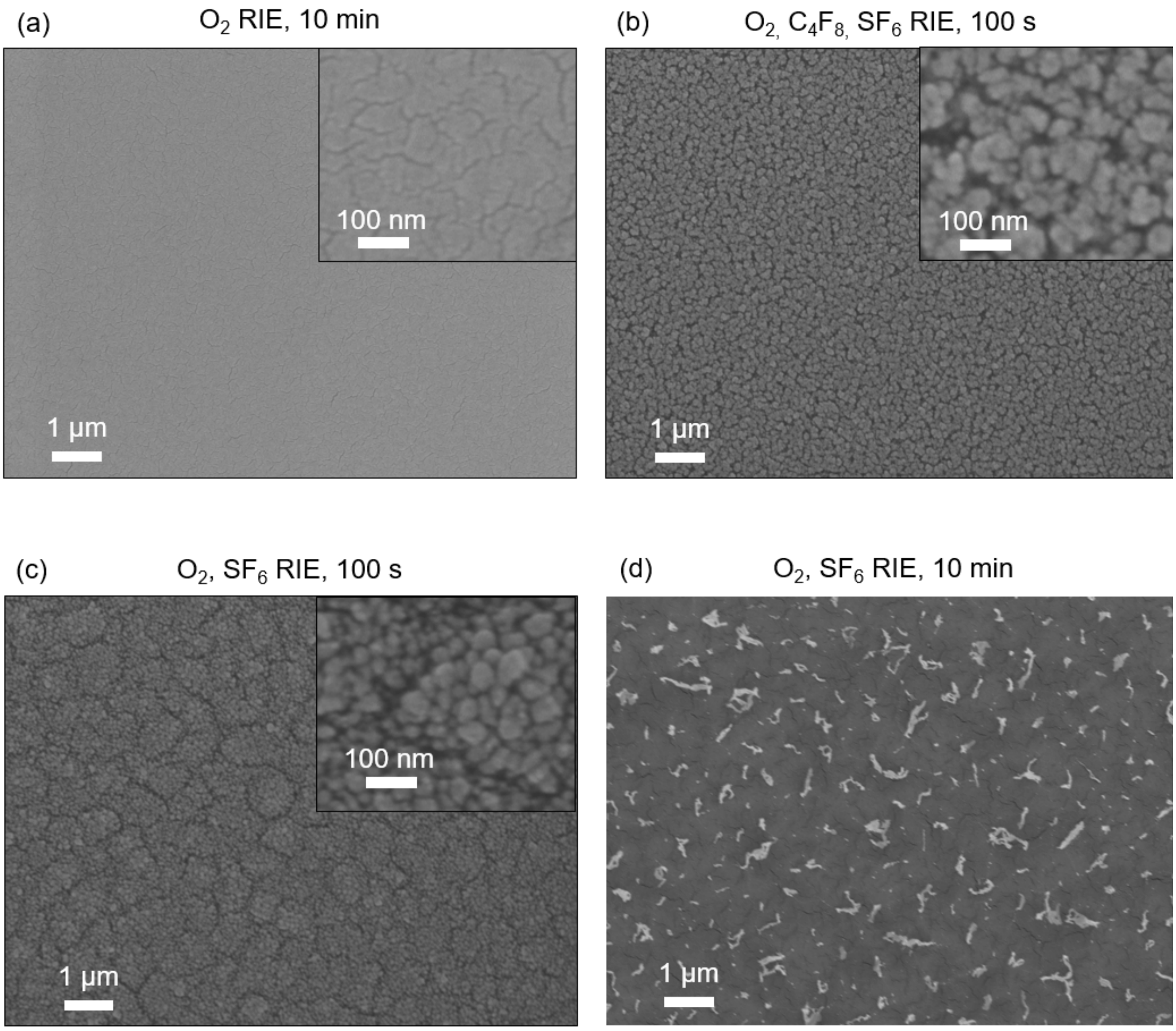

Figure S2. *Surface roughness.* The SEM images show the surface roughness after dry etching of the thiol polymer in an ICP RIE for different etching recipes and times. The specific parameters for each recipe can be found in Table 3 in the main text.
